# What Personality Dimensions May Influence the Risk of Smartphone Addiction in Children?

**DOI:** 10.3390/children12030258

**Published:** 2025-02-20

**Authors:** Stella Conte, Carla Ghiani, Lorenzo Casati, Roberto Truzoli, Eraldo Francesco Nicotra

**Affiliations:** 1 Department of Pedagogy, Psychology and Philosophy, University of Cagliari, 09123 Cagliari, Italy; stella.conte@unica.it (S.C.); carla.ghiani@unica.it (C.G.); 2 Department of Biomedical and Clinical Sciences, University of Milano, 201257 Milano, Italy; lorenzo.casati@unimi.it (L.C.); roberto.truzoli@unimi.it (R.T.)

**Keywords:** new technology, smartphone, addiction, children, personality, BFC, big five, SARCQ, structural equation models (SEMs)

## Abstract

Background: Smartphone usage in school-aged children has increased over the last two decades. This overuse interferes with emotion regulation and interpersonal relationships. The purpose of this work was to analyze the relationship between smartphone addiction risk and personality dimensions in primary school children. Methods: The aim of this research is to verify the percentage of Smartphone Addiction (SA) in a sample of primary school children and to explore the relationship between personality dimensions and SA. The Smartphone Addiction Risk Children Questionnaire (SARCQ) and the Big Five Children (BFC) questionnaire were administered to a sample (N = 94) of children. Results: We found that, in our sample, the percentage of children matching the definition of emotional addiction to smartphones was 16% and that a subgroup of children using smartphones as a transitional object represented 15% of the sample. The correlations between the SARCQ and BFC questionnaire factors showed a significant negative correlation between the “I’m not afraid with you” (INAWY) factor and Friendliness, Conscientiousness, and Openness, showing that children with low socialization capacities are prone to use smartphones as a means to handle negative internal states. In contrast, a positive correlation between the INAWY and the Emotional Instability factors has been observed. For the “Linus’s Blanket” (LB) factor, a significant negative correlation with the Friendliness and Conscientiousness factors was observed. Conclusions: The risk of SA, with the use of smartphones as “emotion-handling tools” or as “transitional objects”, was observed in children with personality dimensions associated with higher risk.

## 1. Introduction

During 2023 in Italy, 91.6% of young people in the 15–17 age group used the Internet every day [1] and smartphones were in first position regarding the devices with the widest rate of diffusion. Within the 11–13 age group, 78.3% of Italian children used the Internet every day, mainly through smartphones. The age at which young people first own or use a smartphone also decreased and, after the COVID-19 pandemic, which forced children to stay at home with no chance of connecting with peers besides virtual means, with increased Internet use to compensate, there has been a significant increase in children aged 6–10 years who use smartphones every day, from 18.4% (2018–19) to 30.2% (2021–22) [2]. This same phenomenon has been observed worldwide, with a general lowering of the age of first contact with mobile devices [3,4]. The use of smartphones has radically changed people’s daily habits, facilitating communication and access to information without constraints on time and place; on the other side, evidence shows an increasing number of individuals who are suffering from problematic smartphone usage [5,6,7]. A great number of studies have underlined the association between the use of smartphones, Internet addiction, and psychiatric disorders, mostly in the area of mood and anxiety [8,9,10,11].

Specifically, in school-age children, Smartphone Addiction (SA) involves negative consequences on daily mental, psychological, physical, and social functioning [12,13] and, like all addiction disorders, it is a complex and multifaceted condition [14,15,16]. SA has been described as the effect of unrestrained smartphone use in one’s daily routine [17], and it comprises symptoms concerning the availability of the device, craving for its access, and scarce positive outcomes in interpersonal relationships [18]. The concept of addiction has been associated with technology since the early years of the Internet’s spread [19], and a recent systematic review provided consistent evidence of neuroanatomical abnormalities in individuals with SA in the gratification circuit and in areas involved in executive functions [20]. In this light, it has been proposed that SA may be supported by typical personality traits known to be related to addiction and dependency [18].

Personality is a manifold construct that features the dynamic progression of integrative characteristics into what the individual will eventually display in terms of social interactions and behavioral outputs [21]. It begins to develop in the early stages of growth, as it is considered to evolve from the infantile temperament, and it is therefore explorable during the developmental age [22]. Personality dimensions have been extensively analyzed in adolescents and young adults, highlighting traits that may play a protective role or act as risk factors, and also their evolution related to a particular kind of Internet use. In particular, some personality dimensions have been reported to be related to SA, including Impulsivity and instant gratification addiction [23,24]; low self-control [25]; low self-esteem; and low Openness [26]. A study focusing on young adults from different countries [27] found that SA is strongly associated with low Conscientiousness and high Neuroticism, and correlates positively with Social Anxiety and Impulsivity. Considering studies that have referenced the Person component of the I-PACE model [14], a recent meta-analysis [28] covering samples of adults and young adults found that Conscientiousness and Neuroticism, respectively, correlate negatively and positively with Smartphone Use Disorder (SUD), highlighting them as risk factors.

The role of personality dimensions regarding SA in children has been less investigated and comprehensive reviews are still scarce [25].

Based on an analysis of the literature, the purpose of this study is to verify the level of SA and the relationship between SA and personality dimensions in children attending primary schools. To investigate personality dimensions, we decided to use the Big Five Children (BFC) questionnaire [29]. Marengo and colleagues provided empirical evidence that the Big Five personality model is useful for understanding individual differences in Smartphone Use Disorder and suggests using it in the assessment stages [28]. To investigate SA, we used the Smartphone Addiction Risk Children Questionnaire (SARCQ), a test recently validated in Italy [30], which, unlike other similar questionnaires, allows one to assess an individual’s ability to handle negative emotions and loneliness, and to test whether they use their smartphone as a transitional object, as has been described by Winnicott [31].

## 2. Materials and Methods

Two self-assessment questionnaires, the Smartphone Addiction Risk Children Questionnaire (SARCQ) [30] and the Big Five Children (BFC) questionnaire [29], were administered, respectively, to verify A) the presence of SA and its severity and B) to assess the relationship between personality dimensions and SA.

The SARCQ [30] evaluates the risk of SA in children. The SARCQ is composed of 11 items, associated with two specific dimensions (the last 5 items are distractors). The complete questionnaire is shown in Appendix A.

The first dimension, named “I’m not afraid with you” (INAWY), is related to handling negative emotions and loneliness. This dimension correlates with the use of smartphones to avoid negative emotions and their use as a relief. It implies escape from reality, stress, and unhappiness [32]. Moreover, it is also related to the use of smartphones instead of dealing with negative emotions such as fear, sadness, and anger. Children increase their smartphone use to compensate for negative feelings due to negative emotions: “I don’t feel them, so they aren’t there!”. This dimension is also related to the “addiction”: children feel bad if they cannot have their smartphone with them. The items correlated to INAWY are “I feel alone if I can’t use my smartphone”, “I get angry if I can’t use my smartphone”, “I use my smartphone instead of doing something else (for example: play, draw, stay with friends)”, “I use my smartphone to get better when I’m sad”, “I feel sad if something bad is happening and I cannot use my smartphone”, and “I go to sleep late because I use my smartphone”.

The second factor, “Linus’s blanket” (LB), is inspired by the well-known cartoon’s character who held a protective blanket. This dimension is related to the use of smartphones in order to not miss one’s parents and friends. Thus, the smartphone becomes a transitional object for the child. This dimension is related to socialization difficulties and avoiding any direct contact with others or to sublimate their absence. LB is correlated with the following items: “I check my smartphone to see if anyone has called or sent me a message”, “I need to keep my smartphone with me to feel more confident”, “When I take my smartphone with me I feel closer to mom and dad”, “When I take my smartphone with me, I feel safer”, and “Mom and dad are calmer when I take my smartphone with me”.

The answers were on a 3-point Likert scale: 1. Never; 2. Sometimes; 3. Always.

The BFC questionnaire [29] measures personality with five major dimensions.

Energy/Extraversion (E): refers to how cheerful and lively the child is and how much they enjoy connecting with other people and being involved in games. Friendliness (F): refers to how sociable, affectionate, empathetic, and able the child is to establish bonds of friendship. Conscientiousness (C): refers to how able the child is to concentrate and make a commitment in a precise and scrupulous way, and the degree to which they feel able to carry out tasks. Emotional Stability (S): refers to what degree the child can manage their emotions and respond to negative situations without becoming discouraged or angry (in the Italian version, this factor is a reverse factor, called “Emotion Instability”). Openness (O): refers to how imaginative, creative, and curious, the child is, and how able they are to find solutions to issues in school or life, providing the basis for problem-solving capacities and lateral thinking, which, in turn, represent high-value resources to cope with negative internal states derived from everyday stressors.

The answers were on a 3-point Likert scale: 1. Never; 2. Sometimes; 3. Always.

## 3. Participants

A sample of 94 children (age: mean = 9 years, SD = ±9.8 months) were recruited. All participants were Caucasian. Recruited participants were all students of four primary schools pertaining to the Cagliari metropolitan area, south Sardinia, Italy, from March 2022 to June 2022. These schools agreed to take part in this research after a general request addressed to all the schools belonging to this district.

## 4. Procedures

All the parents of participants signed the informed consent form. The SARCQ and BFC questionnaire were completed by participants in classrooms with four researchers supervising. Both the children and their parents were informed that data collection and questionnaire answers would be anonymized. Since all the participants were underage, their parents were asked to sign an informative sheet describing the aim of the research, data processing, informed consent, and the use of the data for research purposes. The study was conducted according to the guidelines of the Declaration of Helsinki and approved by the Ethics Committee of the Department of Education, Psychology and Philosophy of University of Cagliari. The research was approved by the Ethics Board of the University of Cagliari (n. 26/17, on 27 June 2017).

## 5. Results

### 5.1. The SARCQ

The mean frequencies and standard deviation (SD), skewness, and kurtosis were calculated for individual items and are shown in Table 1. The skewness and kurtosis were used to evaluate the relevance of the expected normal asymmetry of the SARCQ items in the direction of small or moderate children’s problems. Specifically, the skewness ranged from −0.067 To 1.224 and the kurtosis ranged from −1.521 to 2.093.

Table 2 shows the correlation matrix of SARCQ items.

An exploratory factor analysis to assess the underlying dimensions of the SARCQ and item loading for each dimension was performed. The extraction method was maximum likelihood (see Joreskög, K.G. (1967); Joreskög, K.G. (1969); and Bollen, K.A. (1989) for details) [33,34,35]. The two-factor solution explains 49.30% of the total variance.

Table 3 and Table 4 show the reliability value of each item for the INAWY and for LB factors.

Cronbach’s α for the SARCQ and both the INAWY and LB sub-scales has been reported in Table 5.

**Figure 1:**
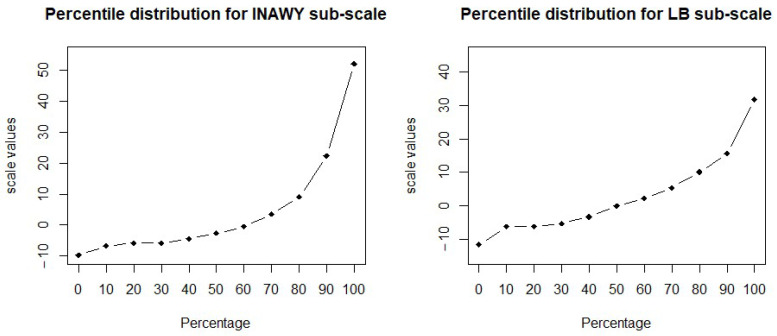
Percentile distributions for both INAWY and LB SARCQs sub-scales.

Table 6 shows the percentage distribution of the weighted scale values for the two factorial dimensions. The percentile distribution for both INAWY and LB sub-scales are shown in Figure 1.

In order to verify the factor structure of the exploratory factor analysis, a confirmatory factor analysis was performed using the statistical program LisRel 8.8 (Joreskög, K.G. (1969, 1970, 1973, 1979)) [33,34,36,37]. Table 7 shows the Lambda-X values of the estimated structural parameters, completely randomized. The solution offered by the confirmatory factor analysis endorsed the two-dimensional structure.

**Figure 2:**
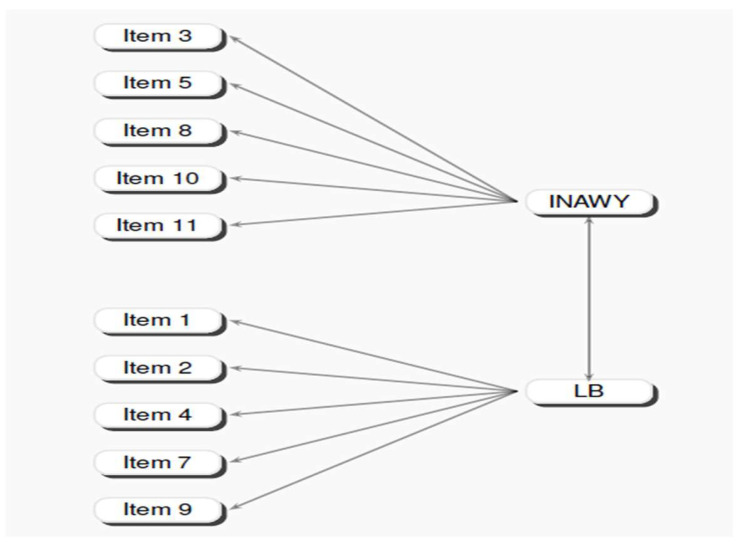
Factor structure of the confirmatory model for SARCQ.

Figure 2 shows the graphic solution of the confirmatory factor model with structural standardized parametric values.

### 5.2. The Structural Equation Model for BFC Questionnaire and SARCQ

**Figure 3:**
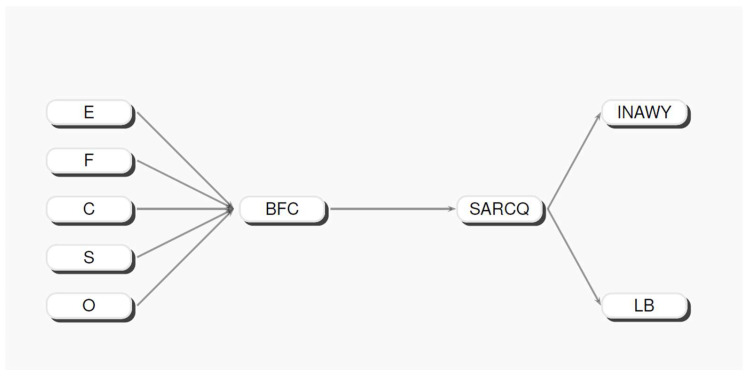
Graphical representation of the structural equation model tested.

First of all, Pearson’s correlation matrix was performed on the INAWY and LB dimensions of the SARCQ and the five factors of the BFC questionnaire. R Version 4.3.2 was used to process the dataset. The correlation structure was then employed and fed into a structural equation model (SEM), in order to discover the strength of the relations existing between the BFC questionnaire, representing the exogeneous dimensions of the model, and the SARCQ dimensions, as the endogenous variables. The parametric solution of the tested model was generated using LisRel 8.8 (see Joreskög, K.G., Sörbom, D. (1988) for details) [38]. The implemented model is shown in Figure 3, whereas the estimated parameters and fit indices are shown, respectively, in Table 8 and Table 9 below. The different typologies of the structural parameters are displayed in the columns; in contrast, the rows show the profile concerning each single indicator of the whole model tested. The analyzed triangular Pearsons’ correlation matrix between the BFC questionnaire and SARCQ is reported in Table 10.

Figure 3 shows the structural equation model solution obtained using the BFC questionnaire as the exogenous latent tract and SARCQ as the endogenous latent tract.

The results of the analyses show that the model may not be rejected in light of the observed values of several Goodness of Fit Statistics, as reported in Table 9.

Moreover, the theta–delta covariance matrix of errors for the five dimensions of the BFC questionnaire was found to be not diagonal, indicating that some measurement error may covariate with some other measurement error. In this case, we found that the error associated with Energy/Extraversion (E) covariated reliably both with Friendliness (F) and Conscientiousness (C) (see Figure 2 for parametric details).

Table 11 shows that the BFC questionnaire’s exogenous indicators may be indirectly associated with the endogenous indicators INAWY and LB of the SARCQ with respect to the indirect effect components.

## 6. Discussion

In recent years, there has been a great increase in smartphone use, being a user-friendly tool with fast access to digital information. They facilitate communication, working, and relationships, but smartphone overuse causes problematic effects in both psychological and physical aspects [39,40,41,42]. The main result of this study is that 1.6 participants out of 10 show signs of SA, in line with a recent nationwide study conducted in South Korea [3]. Moreover, it emerged that the use of smartphones as ”emotion-handling tools” or as “transitional objects” is a concern in particular children that exhibit low Friendliness, low Conscientiousness, and socialization issues [23,43]; these children may favor indirect relationships, experienced through a smartphone, over ones directly experienced in person. It has been proposed that smartphones may be used by individuals with an insecure attachment style [44], such that the anxiety that rises within an interpersonal relationship may be held back by their involvement in virtual interactions. Studies on adult subjects have underlined how this relationship pattern must be challenged, helping patients to develop solid connections in real life rather than on the Internet, in which the relief from attachment anxiety is artificial and unstable [23]. Moreover, socialization issues are prone to isolate people, hampering the creation of strong relationships. It has also been found that loneliness and poor social skills can drive subjects to increase their time on screens [45], and therefore focus on immaterial and mediated interactions instead of talking in person to others. Given that the Internet provides shy individuals with entertainment and various forms of content, SA creates a comfortable environment in which public constraints and difficult situations are easily avoided. Therefore, individuals who suffer from loneliness due to socialization issues might depend on their smartphones more and more, clearing the way for addiction [43]. A feature that represents a receptacle for the negative psychological aspects set forth so far is depressive rumination, highlighted as a key factor in recent works [24,46]: together with Impulsivity, which showed higher loads, depressive brooding proved to be a clear predictor of Internet addiction in a sample of 411 adolescents, being a maladaptive strategy for emotional regulation [47]. In this light, this article provides adjunctive evidence that objects used as emotion-handling tools are prone to represent potential focuses of addictive behaviors.

This research, in fact, has underlined that negative personality dimensions could represent the background of addiction. Thus, primary prevention is crucial. A large body of evidence corroborates the effectiveness of early psychological intervention in preventing the development of mental health issues and aberrant behaviors, especially pertaining to addiction [48,49]. These interventions should be focused on improving personality dimensions such as Friendliness, Emotional Stability, Conscientiousness, and Openness to experiences [50]. Primary prevention is more effective when implemented during childhood and/or early adolescence rather than at later ages, given the impact of SA and the consequences it may cause [51,52]. Internet addiction is often linked to struggles in emotional regulation and it is influenced by various personality traits [53]; these aspects should be taken into consideration when developing effective prevention strategies and treatment. Our results correlate some personality factors, as conceptualized by the Big Five model, with the risk of SA. From a clinical standpoint, in the case of evident SA, the results underscore the appropriateness of Cognitive Behavioral Therapy (CBT) treatment, which is particularly effective in cases of high Neuroticism and emotional regulation issues [54]. Considering preventive perspectives, Theopilus and colleagues [55] identified potential interventions, including the child’s education, parenting strategy, and strategic physical activity. Among the relevant strategies to enhance emotional regulation, we underline improving one’s emotional intelligence, and particularly the ability to regulate one’s emotions; setting boundaries by establishing clear guidelines for Internet use, such as limiting screen time and avoiding online activities during emotional distress; and developing coping skills, encouraging the learning of alternative coping mechanisms, such as physical exercise, creative hobbies, or social interactions. Implementing targeted interventions that focus on enhancing emotional regulation can reduce the need to use smartphones, leading to improved mental well-being. Among the strategies for prevention focused on personality, the development of conscientiousness should be highlighted through increasing self-discipline and organizational skills; engaging in regular physical activity, as this has been shown to mitigate Internet addiction by regulating the neurobiology of the central and autonomic nervous systems (increasing the levels of neurotrophic factors and neurotransmitters, improving overall mental health [56]); and social skills training, focused on balancing online and offline social interactions. Finally, school-based programs promoting positive youth development can be effective in preventing SA, increasing self-control and fostering positive traits [57].

Addressing SA through personality development is a proactive approach that focuses on enhancing traits that can mitigate the risk of excessive online engagement. The application of the SARCQ, specifically developed for children and focusing on the two factors “emotion management” and “smartphone use as a transitional object”, is an innovative element. The results it produces can be used as a specific target both in early treatment and prevention strategies. The identification of young children with low socialization capacities and negative coping strategies and the psychological empowerment of their relational abilities has a paramount importance in their developmental trajectory, avoiding withdrawal from the social dimension and from the enrichment derived by interpersonal relationships. This research is affected by some limitations that may be considered for the design of future studies. First, the small sample size and the homogeneity of the participants’ ethnicity, related to the monocentric design of the study, recruiting children from schools of a single city district, could affect the generalization of the observed results. Second, the transversal framework of the study provides a static overlook, whereas a longitudinal observation may help in understanding the causal relationship between personality dimensions and SA and its evolution over time. The commitment to conducting longitudinal follow-ups has been made, and future observations will clarify the interplay between aging, personality development, and smartphone use, and verify the stability of the observations made. Moreover, smartphone use in children may reflect attitudes or context pertaining to different cultural backgrounds, in terms of parenting styles [58], social consideration of new technologies [59], and their affordability; therefore, it may vary across countries and social classes. Furthermore, as with personality dimensions, it may evolve with aging. As SA may have repercussions in both personal and social contexts [10,11], more detailed observations about problematic smartphone use risk are expected. Considering that social networks in particular have been linked with poor mental outcomes for children in several studies [60], for instance, gathering more details on the specific content (social networks, instant messaging apps, streaming platforms, search engines) accessed by the participants could be profitable for this research topic in the future. In fact, several modalities of smartphone usage have been related to addiction risk, such as entertainment apps, gaming platforms, and social media [61], while educational platforms and apps for writing/composing related to study purposes are less likely linked to SA [62]. Lastly, this study provides evidence for personality dimensions that are related to SA, highlighting the mediating effect that personality may have on strategies to handle internal distress. Further research, focusing on diverse constructs, is required to better understand the causal mechanisms that are implicated in this new kind of addictive behavior.

## Figures and Tables

**Table 1:** Descriptive statistics for the SARCQ.

Descriptive Statistics								
	Mean	S. E. of the Mean	Median	Std. Deviation	Skewness	S. E. of Skewness	Kurtosis	S. E. of Kurtosis
Item 1	2.04	0.038	2	0.823	−0.067	0.113	−1.521	0.225
Item 2	1.62	0.035	1	0.762	0.769	0.113	−0.874	0.225
Item 3	1.3	0.027	1	0.594	1.809	0.113	2.093	0.225
Item 4	1.65	0.034	1	0.742	0.654	0.113	−0.916	0.225
Item 5	1.6	0.033	1	0.722	0.768	0.113	−0.720	0.225
Item 6	1.53	0.029	1	0.638	0.805	0.113	−0.39	0.225
Item 7	1.77	0.037	2	0.807	0.444	0.113	−1.327	0.225
Item 8	1.59	0.033	1	0.721	0.809	0.113	−0.667	0.225
Item 9	1.78	0.038	2	0.821	0.428	0.113	−1.389	0.225
Item 10	1.44	0.031	1	0.665	1.224	0.113	0.234	0.225
Item 11	1.44	0.030	1	0.655	1.212	0.113	0.247	0.225

**Table 2:** Correlation matrix of SARCQ items.

	Item 1	Item 2	Item 3	Item 4	Item 5	Item 6	Item7	Item 8	Item 9	Item 10	Item 11
Item 1	1.000	0.477	0.328	0.523	0.523	0.429	0.299	0.355	0.229	0.338	0.207
Item 2	0.477	1.000	0.481	0.572	0.640	0.473	0.360	0.293	0.217	0.384	0.216
Item 3	0.328	0.481	1.000	0.453	0.428	0.397	0.331	0.262	0.121	0.316	0.193
Item 4	0.523	0.572	0.453	1.000	0.637	0.428	0.361	0.357	0.291	0.489	0.268
Item 5	0.523	0.640	0.428	0.637	1.000	0.431	0.246	0.302	0.217	0.379	0.189
Item 6	0.429	0.473	0.397	0.428	0.431	1.000	0.388	0.415	0.259	0.352	0.290
Item 7	0.299	0.360	0.331	0.361	0.246	0.388	1.000	0.462	0.375	0.505	0.423
Item 8	0.355	0.293	0.262	0.357	0.302	0.415	0.462	1.000	0.412	0.495	0.370
Item 9	0.229	0.217	0.121	0.291	0.217	0.259	0.375	0.412	1.000	0.566	0.502
Item 10	0.338	0.384	0.316	0.489	0.379	0.352	0.505	0.495	0.566	1.000	0.555
Item 11	0.207	0.216	0.193	0.268	0.189	0.290	0.423	0.370	0.502	0.555	1.000

**Table 3:** Squared multiple correlations for INAWY.

Item 3	Item 5	Item 6	Item 8	Item 10	Item 11
0.43	0.59	0.34	0.61	0.62	0.36

**Table 4:** Squared multiple correlations for LB.

Item 1	Item 2	Item 4	Item 7	Item 9
0.41	0.39	0.44	0.69	0.43

**Table 5:** Cronbach’s α values for the SARCQ.

Cronbach’s α	Standardized Cronbach’s α	INAWY Cronbach’s α	LB Cronbach’s α
0.868	0.870	0.849	0.814

**Table 6:** Scale scores.

	Min	10%	20%	30%	40%	50%	60%	70%	80%	90%	Max
INAWY	−9.69	−6.75	−5.87	−5.87	−4.46	−2.62	−0.55	3.57	9.16	22.34	52.13
LB	−11.57	−6.26	−6.26	−5.33	−3.32	−0.049	2.16	5.37	10.07	15.55	31.73

**Table 7:** Confirmatory factor solution (Lambda-X).

LAMBDA-X											
Items	1	2	3	4	5	6	7	8	9	10	11
INAWY			0.65 *		0.77 *	0.58 *		0.78 *		0.79 *	0.60 *
LB	0.64 *	0.63 *		0.66 *			0.83 *		0.66 *		

The bold values are the major inner coherence values for each dimension. All parameters with Lambda-X value were statistically different from zero at *p* = 0.05 (*).

**Table 8:** Structural parameters and error variances for each indicator.

	BFC Questionnaire		SARCQ	
	λ*^x^*	θ^δ^	λ*^y^*	θ^ε^
**E**	0.19	0.96	-	-
**F**	0.78	0.40	-	-
**C**	0.77	0.40	-	-
**S**	−0.53	0.72	-	-
**O**	0.82	0.33	-	-
**INAWY**	-	-	1.00	1.22
**LB**	-	-	−0.25	1.01

**Table 9:** Main Goodness of Fit Statistics.

GFI	AGFI	PGFI	RMR	SRMR	RMSEA	NFI	PNFI	$\chi^{2}$; d.f. = 11	$\frac{\chi^{2}}{d.f.}$
0.98	0.95	0.38	0.23	0.087	0.001	0.97	0.51	7.02	7.02/11

**Table 10:** Correlation matrix of BFC questionnaire and SARCQ.

	E	F	C	S	O	IN	LB
E	1.000	0.293	−0.51	−0.164	0.110	−0.160	0.115
F	0.293	1.000	0.581	−0.436	0.624	0.472	0.188
C	−0.051	0.581	1.000	−0.417	0.667	−0.405	0.034
S	−0.164	−0.436	−0.417	1.000	−0.391	0.313	−0.076
O	0.110	0.624	0.667	−0.391	1.000	−0.466	0.108
IN	−0.160	0.472	−0.405	0.313	−0.466	1.000	0.056
LB	0.115	0.188	0.034	−0.076	0.108	0.056	1.000

**Table 11:** Indirect effects of BFC questionnaire components on SARCQ indicators.

SARCQ				
E	F	C	S	O
−0.11	−0.44	−0.44	0.30	−0.47

## Data Availability

The original contributions presented in this study are included in the article. No new data were created or analyzed in this study. Data sharing is not applicable to this article.

## Appendix A

### Smartphone Addiction Risk Children Questionnaire (SARCQ)

SACRQ is a 13-item questionnaire. It has 11 items measuring smartphone addition:

item 1: “I check my smartphone to see if anyone has called or sent me a message“;
item 2: “I need to keep my smartphone with me to feel more confident”;
item 3: “I feel alone if I can’t use my smartphone”;
item 4: “When I take my smartphone with me I feel closer to mom and dad”;
item 5: “I get angry if I cannot use my smartphone”;
item 6: “I use my smartphone instead of doing something else (for example: play, draw, stay with friends)”;
item 7: “When I take my smartphone with me, I feel safer”;
item 8: “I use my smartphone to get better when I’m sad”;
item 9: “Mom and dad are calmer when I take my smartphone with me”;
item 10: “I feel sad if something bad is happening and I cannot use my smartphone”;
item 11: “I go to sleep late because I use my smartphone”.

The answers were on a 3-point Likert scale: 1. Never; 2. Sometimes; 3. Always.
